# On the crossroads of interdisciplinary medicine in amyloidosis – study protocol for a single-center interdisciplinary registry study

**DOI:** 10.1371/journal.pone.0350084

**Published:** 2026-06-02

**Authors:** Helena Pernice, Gina Barzen, Jakub Piwowarski, Harisa Muratovic-Colic, Anne Pankow, Vera von Landenberg-Roberg, Stephan Bohl, Eva Schrezenmeier, Paul J. Wetzel, Nicolas W. Wieder, Gunnar Fiß, Elisabeth Blüthner, Fabian Knebel, Daniel Messroghli, Stefanie M. Werhahn, Jan Gröschel, Anna-Karina B Maier, Shideh Schönfeld, Christoph Wetz, Jeanette Schulz-Menger, Bettina Heidecker, Axel Nogai, Sebastian Spethmann, Katrin Hahn

**Affiliations:** 1 Amyloidosis Center Charité Berlin (ACCB), Charité Universitätsmedizin Berlin, Germany; 2 Klinik für Neurologie mit Experimenteller Neurologie, Charité Universitätsmedizin Berlin, Germany; 3 Medizinische Universität Lausitz - Carl Thiem, Department of Cardiology, Rhythmology and Angiology, Cottbus, Germany; 4 Deutsches Herzzentrum der Charité, Department of Cardiology, Angiology and Intensive Care Medicine, CCM, Berlin, Germany; 5 Charité – Universitätsmedizin Berlin, corporate member of Freie Universität Berlin and Humboldt-Universität zu Berlin, Berlin, Germany; 6 DZHK (German Centre for cardiovascular research), Partner site Berlin, Germany; 7 Department of Rheumatology and Clinical Immunology, Charité-Universitätsmedizin Berlin, Berlin, Germany; 8 Department of Hematology, Oncology and Cancer Immunology, Charité - Universitätsmedizin Berlin, corporate member of Freie Universität Berlin and Humboldt-Universität zu Berlin, Berlin, Germany; 9 Department of Nephrology and Medical Intensive Care, Charité - Universitätsmedizin Berlin, Corporate Member of Freie Universität Berlin and Humboldt-Universität zu Berlin, Berlin, Germany; 10 Berlin Institute of Health at Charité- Universitätsmedizin Berlin, Berlin, Germany; 11 Medizinische Klinik m.S. Hepatologie und Gastroenterologie, Charité Universitätsmedizin Berlin, Campus Charité Mitte, Berlin, Germany; 12 Klinik für Innere Medizin mit Schwerpunkt Kardiologie, Sana Klinikum Lichtenberg, Berlin, Germany; 13 Deutsches Herzzentrum der Charité, Department of Cardiology, Angiology and Intensive Care Medicine, CVK, Berlin, Germany; 14 Department of Ophthalmology, Charité – Universitätsmedizin Berlin, Berlin, Germany; 15 Klinik für Nuklearmedizin, Charité Universitätsmedizin Berlin, Germany; 16 Helios Clinics Berlin Buch, Department Cardiology and Nephrology, Schwanebecker Berlin, Germany; 17 Charité – Universitätsmedizin Berlin, ECRC a joint institution of Charité and MDC, Berlin, Germany; 18 Deutsches Herzzentrum der Charité, Department of Cardiology, Angiology and Intensive Care Medicine, CBF, Berlin, Germany; 19 Onkologische Schwerpunktpraxis Tiergarten, Berlin, Germany; Shanghai Jiao Tong University, CHINA

## Abstract

**Background:**

Systemic amyloidosis comprises a heterogeneous group of rare diseases characterised by extracellular deposition of misfolded protein fibrils, leading to progressive organ dysfunction. Due to the variability in clinical presentation and course, collection of system-specific and longitudinal data is essential for understanding disease progression, treatment response and patient outcomes. At the Amyloidosis Center Charité Berlin (ACCB), a prospective amyloidosis registry has been established to systematically collect clinical, laboratory, imaging and patient-reported data with the aim of improving the characterization of the diseases and facilitating translational research.

**Methods:**

This is a single-center prospective registry study that enrols patients diagnosed with systemic amyloidosis. The registry includes demographic data, multidisciplinary clinical phenotyping, biomarkers, biobanking, genetic information, imaging studies, and patient reported outcomes. Here, we describe the standardised protocol for diagnostic workup, baseline and longitudinal data collection, and disease-specific follow-up algorithms. Data will be collected digitally in interoperable data formats to ensure shareability in accordance with GDPR-policies.

**Discussion:**

This registry will serve as a resource for characterizing amyloidosis as a rare disease model in a real-world setting and identifying patterns in disease progression and treatment efficacy. By prospectively collecting high-quality longitudinal data, the study aims to generate insights that can inform clinical decision-making, improve risk stratification and support future intervention studies. In addition, the registry enables collaboration in the discovery of biomarkers and new therapeutic approaches. Ongoing analysis of this cohort will provide a basis for the further development of personalised treatment strategies and the improvement of patient care.

**Ethics and dissemination:**

Ethical approval was given by the local ethic committee. Dissemination of data in publications with different scientific observational and correlational questions is planned. **Clinical trial registration**: DRKS00032002

## Introduction

Amyloidosis enco‌‌mpasses a diver‌‌se group of rare diseases characterised by the extracellular deposition of misfolded protein fibrils, which disrupt normal tissue architecture and function [1]. The disease can affect mult‌‌iple organ systems, including the heart, kidneys, liver, nervous system, and gastrointestinal tract, leading to significant morbidity and mortality. Among the systemic forms, light-chain (AL) amyloidosis, transthyretin (ATTR) amyloidosis, and serum amyloid A (AA) amyloidosis are the most common subtypes, each with distinct pathophysiology, clinical presentation, and therapeutic considerations. Despite advances in diagnostic techniques and treatment options, amyloidosis remains a challenging condition due to its heterogeneous nature, delayed diagnosis, and limited data on long-term outcomes [2,3].

Interdisciplinary care is essential for managing rare diseases affecting multiple organ systems. Effective collaboration among specialists, primary care providers, and researchers requires seamless communication and data sharing, which can be hindered by fragmented healthcare systems and incompatible digital platforms. Differing clinical perspectives and treatment priorities can also lead to inconsistencies in patient care. Additionally, logistical barriers such as scheduling conflicts and limited access to specialised expertise may delay diagnoses and interventions. In amyloidosis, the time from first presentation until the diagnosis may take several years, especially in more unusual disease presentations [4]. Addressing these challenges require improved interoperability of health systems, standardised protocols, and integrated digital tools to enhance coordination and optimize patient outcomes. Large-scale, systematically collected clinical data are essential to improving disease characterization, understanding natural history, and optimizing treatment strategies [5]. While previous amyloidosis registries, such as the THAOS study, have provided valuable insights into disease epidemiology and prognosis [6,7], there remains a need for standardised and interoperable, continuous real-world, longitudinal data collection that reflect contemporary diagnostic and therapeutic advancements, allowing collaborative data collection between centers. Such prospective registries play a critical role in identifying prognostic markers, evaluating treatment responses, and supporting the development of personalised management approaches. In this study, we envision in depth standardization and interoperability enabling federated collaboration, expanding the potential of a single-center registry.

### Objectives

To address these gaps, we established a prospective interdisciplinary amyloidosis registry at our university hospital, aiming to systematically collect and analyse clinical, laboratory, imaging, and patient-reported outcomes from individuals diagnosed with systemic amyloidosis. This digital registry serves as a resource for both observational and translational research, fostering collaborations in biomarker discovery, disease modelling, and therapeutic innovations. For rare diseases like amyloidosis, digital registries improve data accuracy, enable real-time sharing, and support predictive models for disease progression. They also foster international collaboration, broadening our understanding of rare conditions. Our prospective registry study leverages digital innovations to collect high-quality data, advance clinical research, and improve patient care while ensuring a secure and sustainable framework for collaborative amyloidosis research. The present study describes the design, methodology, objectives and IT-specifications of this registry and provides a framework for future research and clinical applications. Importantly, this registry study incorporates individualization of data collection based on clinical care, integrating data collection smoothly into clinical care processes. Here, we roll out an example for time-efficient, care-based data collection for present and future interoperable and big-data analysis.

### Study design

This study is a single-center, observational, prospective cohort study (protocol version 1, 03.2020).

## Methods and analysis

### Study setting

The interdisciplinary Amyloidosis Registry is hosted by the Charité – Universitätsmedizin Berlin and embedded in the specialised Amyloidosis Center Charité Berlin (ACCB), which encompasses several medical specialties, including neurology, cardiology, hematology-oncology, nephrology, gastroenterology, radiology, nuclear medicine and ophthalmology. The study was approved by local ethical committee of the Charité – Universitätsmedizin Berlin and was registered under the identifier DRKS00032002 at the German Registry of Clinical Studies (DRKS – Deutsches Register Klinischer Studien). Participants are recruited via the ACCB outpatient clinic as well as from referring physicians with suspected or confirmed diagnosis of amyloidosis. Referring physicians are openly invited to present cases at the ACCB case conference (contact via amyloidosis-center@charite.de) and scheduled for study inclusion and data collection in the corresponding ACCB specialty outpatient clinic. The study is purely observational and collects longitudinal data from patients with amyloidosis or suspected amyloidosis (until exclusion of amyloidosis). Primary presentation of patients with amyloidosis requires the identification of [1] organ manifestation, [2] amyloid type, and [3] therapeutic approach. Since many types of amyloidosis can affect several different organ systems, patients may present at different specialists, which can impose challenges in the structured documentation and continuation of workflow. To address this, patient cases are discussed in weekly case conferences in an interdisciplinarity fashion, always including at least one specialised cardiologist, neurologist, and hematologist, as well as a nuclear medicine specialist, and specialists depending on the respective organ manifestation of patients discussed. In the case conference, the diagnosis is either confirmed and treatment as well as follow-up procedure depending on the respective diagnosis decided, or further diagnostics and a new case conference are planned.

### Study status

Ongoing (study start: 03.04.2020; study duration: at least 10 years; end date: 03.04.2030 or later; expected completion of participant recruitment: 2030 or later, expected results from study: 2030 and earlier for sub-study results). On March 31^st^ 2026, recruitment included a total of 849 patients.

### Eligibility criteria

#### Inclusion criteria.

1. Age ≥ 18 years
2. Patients with suspected or diagnosed amyloidosis (any of the below)
  a. Clinically suspected diagnosis resulting from imaging or laboratory parameters
  b. Histomorphological diagnosis of amyloidosis or positive DPD scan
  c. Genetically confirmed TTR mutation
3. Voluntary signing of informed consent (including opt-in options for collection of biological specimens)

#### Exclusion criteria.

1. Age < 18
*2.* Lack or inability for voluntary signing of informed consent

### Interdisciplinary data collection

This structure enables comprehensive patient evaluation and multidisciplinary collaboration. The registry is designed to systematically collect longitudinal clinical, laboratory, imaging, and patient-reported data.

### Ethics approval and consent to participate

The study was approved by the Ethics Committee of Charité—Universitätsmedizin Berlin (Ref. EA1/014/20). Written informed consent is obtained from all participants prior to enrolment. The consent process covers (i) the scope of data and sample collection; (ii) optional written consent for long-term biobanking (serum/EDTA plasma, aliquoted and stored) and written consent for any genetic testing relevant to amyloidosis; (iii) permission to publish de-identified aggregate results; and (iv) the right to withdraw at any time without impact on clinical care, including options for destruction of stored samples and cessation of further data use. Participants are explicitly informed that only future use of data or biospecimen collected during the study is stopped upon withdrawel, as participant records, all associated data and biospecimen will be eliminated. However, data already analysed or published cannot be removed, biological samples already distributed or used cannot be eliminated, and clinical records including results from study analyses, such as genetic findings, cannot be removed from existing medical records.

Genetic counselling is offered to all participants undergoing genetic testing; if a pathogenic variant is identified, counselling addresses implications for at-risk relatives. Incidental findings of clinical significance (imaging, genetic, or laboratory) are returned according to predefined clinical governance procedures, with appropriate referral and follow-up.

### Study Protocol

**Fig 1:**
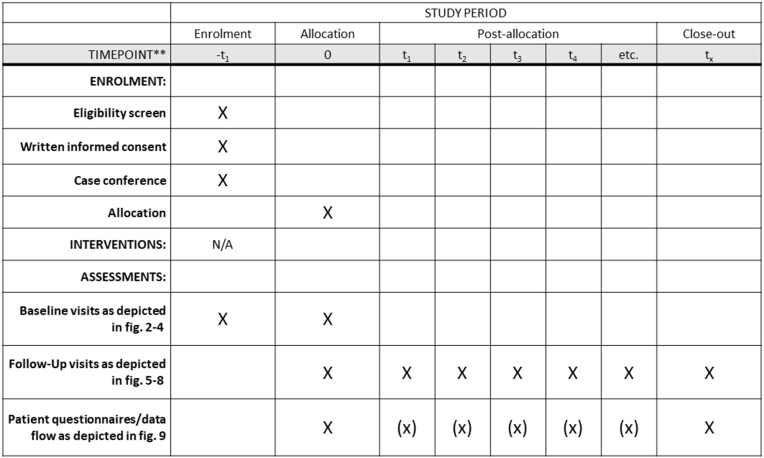
SPIRIT schedule. No interventions are planned as this is a purely observatory study. Timepoints as depicted as t1-t4, etc. are defined in respective figures (as stated in left column) depending on baseline disease. Assessment datasets are defined in Figs 2-8. Core datasets are indicated as X; extended datasets are indicated by the symbol **(x)**.

This study aims at understanding real-world disease course, treatment effects, and quality of life of patients affected with systemic amyloidosis in an interdisciplinary setting. In order to achieve a broad understanding of organ involvement and outcomes, system-specific outcome measures were chosen to record longitudinal patient data dependent on organ involvement. For each specialty, we defined a standardised dataset to enable research and further collaboration efforts depending on the diagnosis. Although patients may present at any discipline when amyloidosis is first suspected, all patients undergo detailed cardiology and neurology examinations. Within the cardiology and neurology departments, informed written consent is obtained, and patients are enrolled in the registry study using a web-based database (Tbase) [8], that has been modified to reflect the needs of the ACCB. Tbase has been established, facilitating the easy entry of key data. This includes basic information such as demographics, height, weight, (suspected) diagnosis, (primary) organ manifestation, and therapy. Additionally, structured medical history and examination data are documented digitally and numerically to enable structured data analysis. Laboratory, imaging, and other ancillary data are automatically integrated from the hospital information system. Fig 1 presents an overview of enrolment and study visits.

### Baseline data collection ATTR amyloidosis

**Fig 2:**
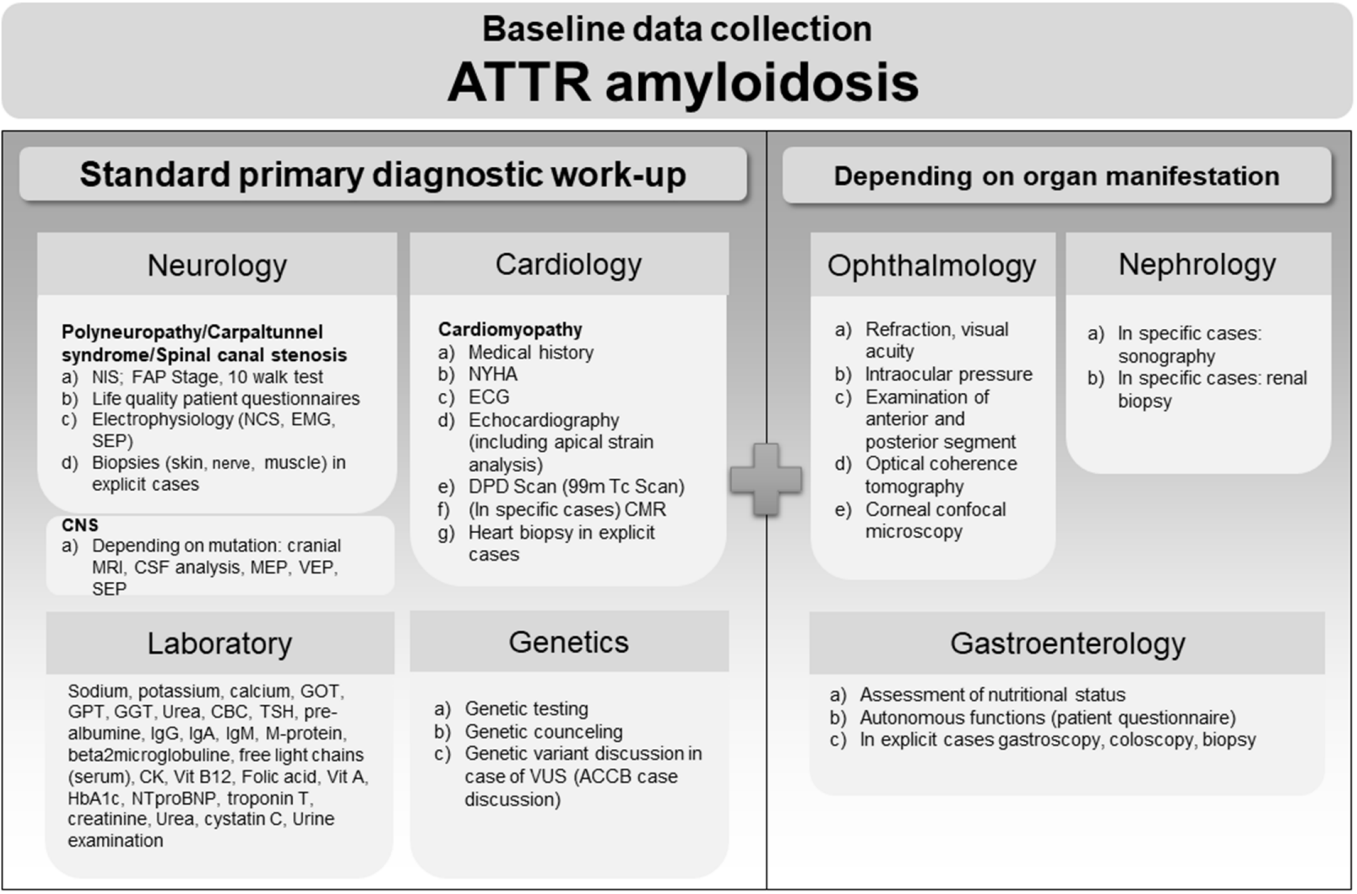
Primary diagnostic workup of patients with ATTR amyloidosis at the ACCB. Baseline examinations after diagnosis (left) as well as additional examinations depending on organ manifestation (right) are depicted for each specialty. NIS, neuropathy impairment score; FAP stage, family amyloid neuropathy stage, NCS, nerve conduction studies; EMG, electromyography, SEP, sensory evoked potentials; MRI, magnetic resonance imaging; CSF, cerebrospinal fluid examination; MEP, motor evoked potentials; VEP, visual evoked potentials; GOT, aspartate aminotransferase; GPT, glutamate pyruvate alanine aminotransferase; GGT, gamma-glutamyl transferase; CBC, complete blood count; TSH, Thyroid Stimulating Hormone; IgG, immunoglobulin G; IgA, immunoglobulin A; IgM, immunoglobulin M; CK, creatine kinase; Vit B12, vitamin b12; Vit A, vitamin A; HbA1c, glycated hemoglobin; NTproBNP, N-terminal pro B-type natriuretic peptide; NYHA, New York Heart Association heart failure stage; ECG, electrocardiogram; DPD, 99mTc-3,3-diphosphono-1,2-propanodicarboxylic acid; CMR, cardiac magnetic resonance imaging; VUS, variant of unknown significance.

Patient with suspected ATTR amyloidosis always undergo cardiological, neurological, and genetic examinations. As depicted in Fig 2, neurological examination includes detailed cognitive, sensory, and motor examinations with quantification using the Neuropathy Impairment Score (NIS). Nerve conduction studies (NCS), electromyography (EMG), and sensory evoked potentials are performed for baseline definition of neuropathy or dysfunction of central conduction due to, i.e., spinal canal sten‌‌osis. Further examination of affection of the central nervous system is performed depending on the patient’s symptoms as well as the underlying genotype (in case of hereditary ATTR (ATTRv) amyloidosis). Likewise, biopsies are only performed in specific cases, e.g., variants of unknown significance (VUS) and uncertainty regarding the affection of the peripheral nervous system. The basic cardiological examinations include a medical history, symptoms according to the New York Heart Association definition (NYHA stage), an electrocardiogram (ECG), transthoracic echocardiography including strain analysis. All patients receive a clinical examination (including additional tests such as the 6-minute-walk-test in selected cases), blood pressure measurement, and a technetium-99m (99mTc) and 3,3-diphosphono-1,2-propandicarboxylic acid (DPD) scan. If high free serum light chains or monoclonal gammopathy of unclear significance (MGUS) are detected or in the case of non-conclusive findings, a biopsy of an involved organ is performed. Cardiac magnetic resonance imaging (CMR) including cine imaging, parametric mapping and late Gd enhancement assessment is also performed in selected cases. CMR is evaluated in a core laboratory setting blinded for clinical information. Laboratory profiles are taken either during cardiology or neurology visits and include parameters of cardiac, renal, hepatic function, as well as parameters to detect important differential diagnoses for peripheral neuropathies such as long-term blood sugars and vitamins. Likewise, genetic testing is performed in all patients with suspected ATTR amyloidosis after written informed consent. Genetic counselling in case of detection of TTR mutation also includes discussion of testing of unaffected first-degree family members and VUS. Unless clearly identified as benign from the literature, patients carrying VUS are included in the same observational examinations as patients carrying pathogenic mutations. Depending on organ manifestation, individual symptoms, and genetic variant, all (potentially) affected organ systems are systematically examined as depicted in Fig 2, right side. Ophthalmologic examination includes refraction, visual acuity, intraocular pressure, examination of anterior and posterior compartments, as well as optical coherence tomography. Nephrological examination includes expanded laboratory testing, while biopsies are only performed in cases with unclear renal involvement or concurring etiologies. Gastroenterological testing includes questionnaires for autonomous function, as well as endoscopy in cases of severe involvement, including deep colon biopsies for proof of amyloid depositions. Hematological testing includes interpretation of laboratory findings in case of detection of an MGUS and performing a bone marrow biopsy for detection of amyloidosis and for diagnosis of plasma cell dyscrasias.

### Baseline data collection AL amyloidosis

**Fig 3:**
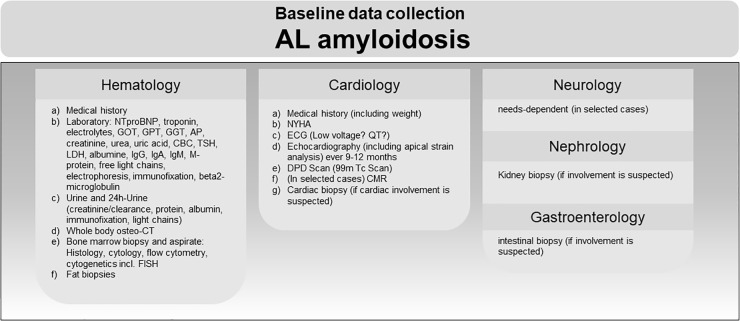
Primary diagnostic workup of patients with (suspected) AL amyloidosis at the ACCB. Examinations after diagnosis (left) as well as additional examinations depending on organ manifestation (right) are depicted for each specialty. Abbreviations: NTproBNP, N-terminal pro B-type natriuretic peptide; GOT, aspartate aminotransferase; GPT, glutamate pyruvate alanine aminotransferase; GGT, gamma-glutamyl transferase; CBC, complete blood count; TSH, Thyroid Stimulating Hormone; IgG, immunoglobulin G; IgA, immunoglobulin A; IgM, immunoglobulin M; CK, creatine kinase; Vit B12, vitamin b12; HbA1c, glycated hemoglobin; osteo-CT, quantitative computed tomography; FISH, fluorescent in situ hybridization; NYHA, New York Heart Association heart failure stage; ECG, electrocardiogram; DPD, 99mTc-3,3-diphosphono-1,2-propanodicarboxylic acid; CMR, cardiac magnetic resonance imaging.

Patients with AL amyloidosis always present to hematology and cardiology outpatient clinics. As depicted in Fig 3, we include structured medical history, laboratory testing, urine testing, and whole-body-CT scan for detection of osteolysis. Whenever an underlying malignancy is suspected, bone marrow aspirates and biopsies including histology, cytology, flow cytometry and cytogenetics are performed. Since systemic AL-amyloidosis often affects the heart [9], cardiological screening is always performed, including NYHA stage, ECG, echocardiography, DPD-scintigraphy. For confirmation of AL-amyloidosis, a histologic confirmation of the subtype is mandatory. Therefore, a biopsy of an affected organ is performed if histologic confirmation is lacking [10]. Depending on organ manifestations suspected clinically or during laboratory screening, neurological, nephrological, or gastroenterological data is collected by the according specialist.

### Baseline data collection AA amyloidosis

**Fig 4:**
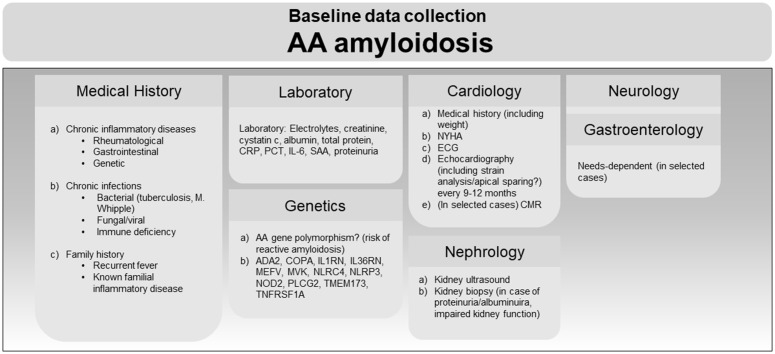
Baseline diagnostic workup of patients with (suspected) AA amyloidosis at the ACCB. Examinations are depicted depending on data type (left) and specialties involved (right). CRP, c-reactive protein; PCT, procalcitonin; IL-6, interleukin 6; SAA, serum amyloid A; ADA2, adenosin desaminase 2; COPA, Coat Protein Complex I Subunit Alpha; IL1RN, interleukin-1 receptor antagonist protein; IL36RN, interleukin-36 receptor antagonist protein; MEFV, mediterranean fever; MVK, mevalonate kinase; NLRC4, NLR family CARD domain containing 4; NLRP3, NLR family pyrin domain containing 3; NOD2, nucleotide binding oligomerization domain containing 2; PLCG2, phospholipase C gamma 2; TMEM173, stimulator of interferon response cGAMP interactor 1/STING1; TNFRSF1A, TNF receptor superfamily member 1A; NYHA, New York Heart Association heart failure stage; ECG, electrocardiogram; CMR, cardiac magnetic resonance imaging.

AA amyloidosis is caused by the extracellular deposition of serum amyloid A (AA) protein, typically in the context of chronic inflammation due to autoimmune, autoinflammatory, infectious, or neoplastic condition [11]. In addition, recent studies suggest that gain-of-function mutations in the *SAA* promoter region may represent a genetic contributor to disease susceptibility and progression [12]. Patients most commonly present to nephrology with nephrotic-range proteinuria and/or impaired kidney function [13]. Medical history focuses on underlying rheumatological disease (autoimmune or autoinflammatory), gastrointestinal symptoms, genetic background, and other potential etiologies such as chronic infections or immunodeficiencies. The diagnosis of AA amyloidosis is confirmed by histological detection of AA amyloid deposits. Selection of the biopsy site is guided by the affected organ and the invasiveness of the procedure. In case of proteinuria or impaired kidney function, a kidney biopsy is recommended to confirm the diagnosis and exclude alternative treatable renal diseases.[14]. Systemic organ involvement should be assessed clinically and through laboratory testing as summarised in Fig 4. Proteinuria is often the earliest and most readily detectable manifestation of AA amyloidosis, reflecting renal involvement. However, systemic organ involvement is common, with frequent involvement of the gastrointestinal tract (including liver and spleen), the peripheral nervous system (e.g., neuropathy), and the heart (e.g., restrictive cardiomyopathy) [15]. Baseline data is collected from all organ system suspected to be involved during baseline screening in the rheumatological outpatient clinic.

### Other amyloidosis types

Other amyloidosis types, such as Gelsolin amyloidosis, undergo system-specific examinations following the scheme for ATTRv amyloidosis. Here, individually defined further data is collected depending on the organ systems involved. Types of amyloidosis that are limited to single organs (e.g., cerebral amyloid angiopathy or Alzheimer’s disease) are not included into the registry.

### Interdisciplinary case conference

After collection of clinical data from cardiology, neurology, as well as any other potentially relevant discipline as outlined above, interdisciplinary board discussions take place in a weekly manner to collect and confirm diagnosis as well as treatment initiation and follow-up. This digital meeting allows internal as well as external physicians to present cases for interdisciplinary discussion and results in a protocol including suggestions for next steps. The protocol is added to the digital registry and communicated to the patient by the primary responsible ACCB physician, either in writing or by phone. The primary respobsible physician is assigned based on the patient’s most prominent organ manifestation. Below, the outline of the interdisciplinary protocol is depicted.

- Date of conference
- Participants: In every case conference, at least one representative of the following specialties with expertise in amyloidosis participates: Neurology, Cardiology, Hematology, Radiology (Nuclear medicine). If available or requested for special questions, representatives of Nephrology, Ophthalmology, Gastroenterology, Rheumatology and others.
- Case discussion:
  ◦ Patient identifier: Pseudonym or initials/birthdate
  ◦ Clinical context: Short depiction of most relevant symptoms and clinical findings leading to suspected diagnosis or amyloidosis or current question to be discussed.
  ◦ Current question: Confirmation of diagnosis, therapy initiation, discussion of side effects or other reason for case presentation
  ◦ Plan: Short summary of consented procedure including responsibilities.

Other topics to be discussed (organizational, research related etc.)

### Criteria for study discontinuation

Data collection and observation in the ACCB Registry study is terminated if the diagnosis of amyloidosis is excluded.

### Participant timelines/follow-up

Patient visits are dependent on individual disease course and underlying amyloidosis subtype. For each subtype, we predefined visit frequencies as depicted in the following sections.

### ATTRv amyloidosis follow-up

**Fig 5:**
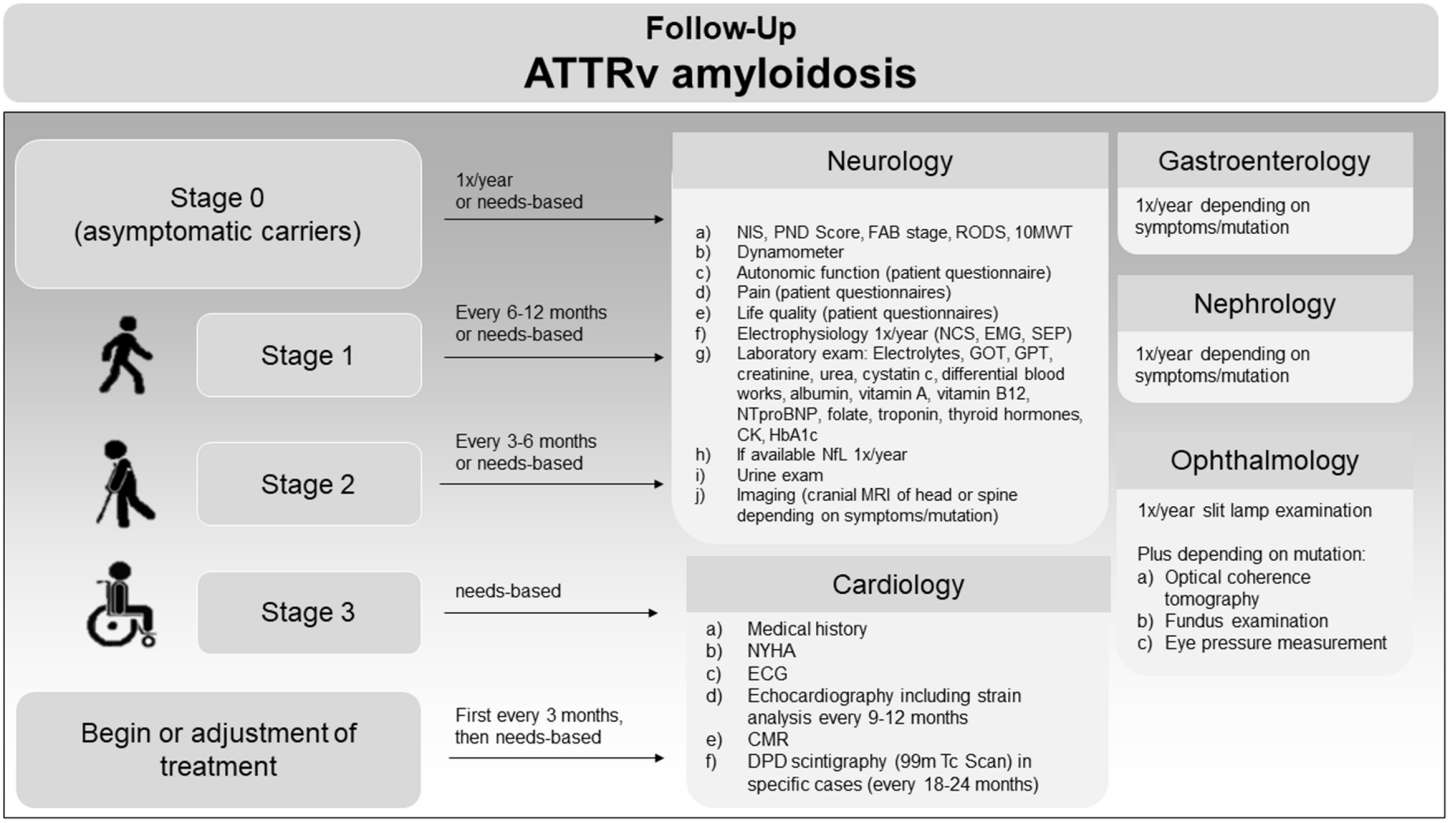
Follow-up of patients with confirmed ATTRv amyloidosis at the ACCB. Left: Disease severity based on FAP stage, arrows indicate the time between follow-up visits, the charts on the right-hand side indicate the examinations planned at each follow-up visit depending on each specialty. Core datasets encompass NIS [a] for neurology and medical history/NYHA [a-b] for cardiology. NIS, neuropathy impairment score; FAP stage, family amyloid neuropathy stage; RODS, Rasch modified Overall Disability Score; 10MWT, 10 meter walk test; NCS, nerve conduction studies; EMG, electromyography, SEP, sensory evoked potentials; MRI, cranial magnetic resonance imaging; NfL, neurofilament light chain; CMR, cardiac magnetic resonance imaging; CSF, cerebrospinal fluid examination; MEP, motor evoked potentials; VEP, visual evoked potentials; GOT, aspartate aminotransferase; GPT, glutamate pyruvate alanine aminotransferase; GGT, gamma-glutamyl transferase; CBC, complete blood count; TSH, Thyroid Stimulating Hormone; IgG, immunoglobulin G; IgA, immunoglobulin A; IgM, immunoglobulin M; NTproBNP, N-terminal pro B-type natriuretic peptide; CK, creatine kinase; Vit B12, vitamin b12; Vit A, vitamin A; HbA1c, glycated hemoglobin; NfL, serum neurofilament light chains; MRI, magnetic resonance imaging; NYHA, New York Heart Association heart failure stage; ECG, electrocardiogram; CMR, cardiac magnetic resonance imaging; DPD, 99mTc-3,3-diphosphono-1,2-propanodicarboxylic acid; VUS, variant of unknown significance.

After diagnosis and staging of patients, we developed a stage-specific follow-up pattern, as described in Fig 5. The minimal core dataset for ATTRv amyloidosis follow-up includes the NIS for neuropathy and medical history/NYHA for cardiology (Fig 5, [a], NIS, in neurology; medical history and NYHA stage [a] and [b] in cardiology).

### ATTRwt amyloidosis follow-up

**Fig 6:**
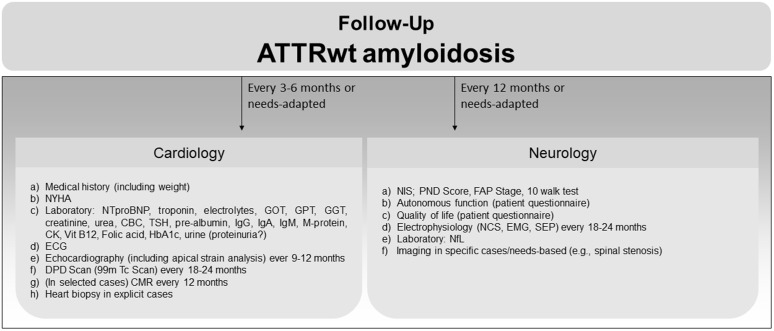
Follow-up of patients with confirmed ATTRwt amyloidosis at the ACCB. Arrows indicate time between follow-up visits; charts depict examination performed at each visit depending on the specialty. Core datasets encompass medical history/NYHA [a-b] for cardiology and NIS [a] for neurology. NTproBNP, N-terminal pro B-type natriuretic peptide; GOT, aspartate aminotransferase; GPT, glutamate pyruvate alanine aminotransferase; GGT, gamma-glutamyl transferase; CBC, complete blood count; TSH, Thyroid Stimulating Hormone; IgG, immunoglobulin G; IgA, immunoglobulin A; IgM, immunoglobulin M; CK, creatine kinase; Vit B12, vitamin b12; Vit A, vitamin A; HbA1c, glycated hemoglobin; ECG, electrocardiogram; DPD scan, 99mTc-3,3-diphosphono-1,2-propanodicarboxylic acid scintigraphy; CMR, cardiac magnetic resonance imaging; NIS, neuropathy impairment score; PND score, polyneuropathy disability score; NCS, nerve conduction studies; EMG, electromyography, SEP, sensory evoked potentials; NfL, serum neurofilament light chains.

Since wildtype ATTR (ATTRwt) amyloidosis primarily affects the heart, follow-up visits are based on disease severity of cardiomyopathy. However, neuropathy has also been described as an increasingly prominent feature of ATTRwt amyloidosis. We therefore recommend a neurological follow-up every 12 months. Detailed examinations performed are depicted in Fig 6. The minimal core dataset for ATTRwt amyloidosis follow-up includes the NIS for neuropathy and medical history/NYHA for cardiology (Fig 6, [a] and [b] in cardiology, and [a], NIS, in neurology).

### AL amyloidosis follow up

**Fig 7:**
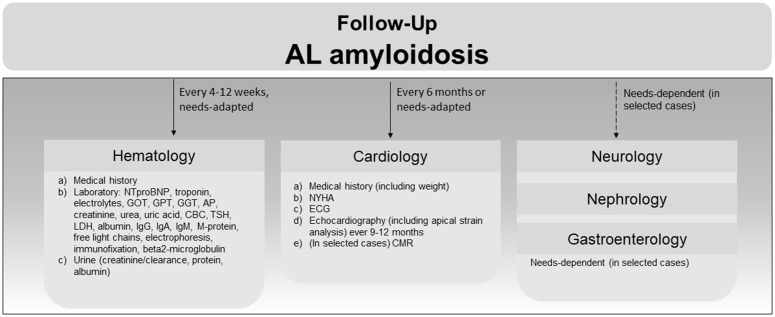
Follow-up of patients with confirmed AL amyloidosis at the ACCB. Arrows indicate time between follow-up visits; charts depict examination performed at each visit depending on the specialty. Core datasets encompass medical history [a] for hematology and medical history/NYHA [a-b] for cardiology. NTproBNP, N-terminal pro B-type natriuretic peptide; GOT, aspartate aminotransferase; GPT, glutamate pyruvate alanine aminotransferase; GGT, gamma-glutamyl transferase; AP, alkaline phosphatase; CBC, complete blood count; TSH, Thyroid Stimulating Hormone; LDH, lactate dehydrogenase; IgG, immunoglobulin G; IgA, immunoglobulin A; IgM, immunoglobulin M; NYHA, New York Heart Association heart failure stage; ECG, electrocardiogram; CMR, cardiac magnetic resonance imaging.

Depending on treatment schemes, AL amyloidosis patients are followed up every 2 weeks initially, and every 3 months in stable disease, aligning with previous studies [16]. Hematological visits include collection of data on clinical symptoms, laboratory testing, and urine testing. Cardiac follow-up is performed at least every 6 months or as required, and includes clinical symptoms, ECG, echocardiography, and, if, CMR. Depending on further organ manifestations, neurological [17,18], nephrological [19], and/or gastrointestinal [20] data collections are performed as depicted in Fig 7. The minimal core dataset for AL follow-up is composed of structured medical history/NYHA (Fig 7, [a] in hematology and cardiology).

### AA amyloidosis follow up

**Fig 8:**
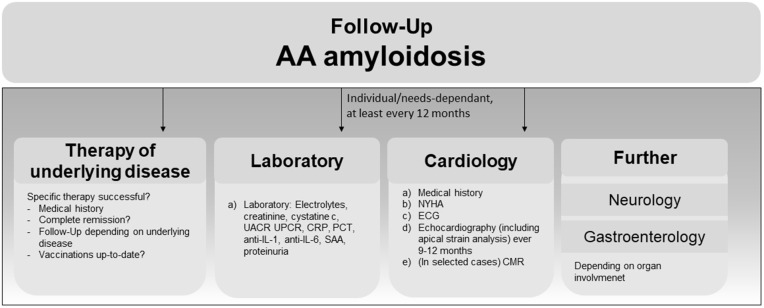
Follow-up of patients with confirmed AA amyloidosis at the ACCB. Arrows indicate time between follow-up visits; charts depict examination performed at each visit depending on the specialty. Core datasets encompass medical history for therapy of underlying disease, and medical history/NYHA [a-b] for cardiology. UACR, urine albumin-creatinine ratio; UPCR, urine protein creatinine ratio; CRP, C-reactive protein; PCT, procalcitonin, anti-IL-1, anti-interleukin 1; anti-IL6, anti-interleukin 6 SAA, serum amyloid A; NYHA, New York Heart Association heart failure stage; ECG, electrocardiogram, CMR, cardiac magnetic resonance imaging.

AA amyloidosis can have various clinical presentations and disease courses, depending on the underlying disease [14]. Follow-up is therefore generally performed on an individual basis but includes structured data collection at least yearly as shown in Fig 8. The minimal core dataset for AA amyloidosis follow-up consists of a structured medical history/NYHA (Fig 8, Medical history; [a] in cardiology).

### Data collection and management

After patient selection and written informed consent, upon registration in our research database (Tbase), each participant is assigned an ongoing number as pseudonymization code. Data is stored using the digital interoperable Tbase platform developed in the clinic as part of research efforts. Keys for patient re-identification is stored within the Tbase platform only accessible by the research personal. The Tbase research database is located within the secure environment of Charité – Universitätsmedizin Berlin, featuring encryption during transmission and storage, role-based access control, and audit logs.

**Fig 9:**
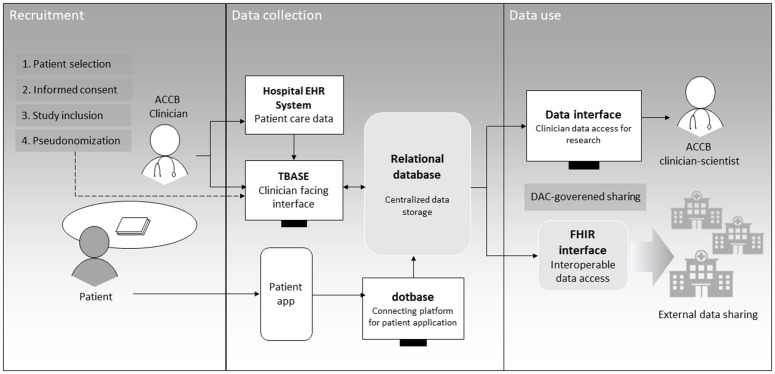
Data flow and technical infrastructure at ACCB. The left side depicts data collection by the physician at study visits or by the patient, and data flow to the relational database (grey box in the middle), which integrates data from the dotbase platform into the tbase platform. The right side depicts data use either by the local researcher or data sharing via the interoperable FHIR format. EHR, electronic health record; FHIR, Fast Healthcare Interoperability Resources; DAC, Data Access Committee.

To collect patient reported outcomes (PROMs) more efficiently, a patient facing smartphone application called dotbase (a digital medical workspace designed for clinical environments, dotbase medical GmbH, Berlin, Germany. Available at: https://dotbase.org) is used, allowing collection of questionnaires from outside of the clinic. Third-party digital tools (dotbase, Tbase) are based on specific collaboration agreements and technology is hosted in Germany. Data access is completely restricted to and hosted by Charité – Universitätsmedizin Berlin. All data access is logged and changes are trackable. Patient recruitment, data flow and distribution are visualised in Fig 9. During clinical visits, physicians directly input data into the electronic database (Tbase). Here, data from the electronic health record (EHR), e.g., laboratory results, imaging, and functional examinations, are included automatically. Data items were selected according to clinical relevance and agreed upon core data sets. The resulting information is combined with patient reported outcomes and additional information such as type of referring physician or date of earliest symptoms.

Data stored in this system can be extracted into tabular format by researchers and physicians via standardised interfaces into common analysis software. Additionally, data can be extracted in the Fast Healthcare Interoperability Resources (FHIR) format [21], or in the form of Human phenotype ontology (HPO) [22] codes [22] per patient. Encoding information in FHIR format allows for quick interoperability with other disease registries. Most data items that are collected inside the registry software are additionally connected to HPO definitions. For each patient, a list of HPO codes, including time domain information, that describes the phenotype and clinical presentation in a standardised way, including ORPHAcodes, can be created. Data sharing is governed by a Data Access Committee (DAC). The DAC consists of the clinical lead (principal investigator), a statistician, and a member of the Ethics board upon request. Internal data access is role based (Role Based Access Control, RBAC). External data access can be requested via email to amyloidosis-center@charite.de and is evaluated by the DAC regarding scientific merit, feasibility, and privacy safeguards. Quality control and periodic data audits are performed bi-yearly and include range checks, detection of duplications, and revision of inter-rater reliability.

We furthermore plan to create an automated connection between the registry database and the data integration center (DIZ) of the university medical center, establishing a direct link between the existing data sharing infrastructure of German university hospitals, as part of the “Netzwerk Universitätsmedizin” (NUM) initiative [23]. This will make data gathered in this registry available to other researchers using the “Forschungsdatenportal Gesundheit” (FDPG) online portal [24], for data privacy ensuring decentralised analysis.

### Biobanking

For biobanking, serum and EDTA blood samples are collected at the first visit, as well as every 12 months. Serum samples are centrifuged at 2,500 g for 10 minutes, both serum and EDTA are distributed into 250µl aliquots and stored at −80°C at the Central Biobank (ZeBanC) for up to 30 years post study inclusion. Additionally, any tissue biopsies taken are stored at the ZeBanC after written informed consent. Access to biospecimen by written request has to be individually granted by the principal investigator. Sample destruction in case of participant withdrawal is performed upon written instruction by the study team.

### Statistical analysis plan

We prespecify the following primary endpoints: [1] overall survival from registry enrolment; [2] organ-specific progression (e.g., incident heart failure, renal failure) defined by laboratory, imaging, and electrophysiological standard clinical criteria (e.g., N-terminal pro B-type natriuretic peptide or reduction of ejection fraction in echocardiography for heart failure, creatinine clearance for renal failure, neurofilament light chains and nerve conduction studies for neuropathy progression, and further as detailed in the subtype-specific sections in the Study Protocol); and [3] longitudinal quality-of-life scores. Secondary endpoints include subtype-specific trajectories and treatment-specific outcomes. Survival endpoints will be calculated using Kaplan–Meier and Cox models; when competing risks are relevant (e.g., cause-specific events), Fine–Gray models will be used. Longitudinal continuous outcomes (biomarkers, PROMs) will be analysed with linear mixed-effects or generalised estimating equations. For comparative effectiveness questions, we will adjust for confounding using multivariable regression and propensity score methods (matching/weighting); the a priori covariate set includes age, sex, subtype, baseline stage/organ involvement, and key comorbidities (DAG-informed).

Missing data will be addressed by multiple imputation by chained equations (MICE) under a MAR assumption; results will be pooled with Rubin’s rules and compared with complete-case analyses in sensitivity checks. We control multiplicity for primary endpoints (e.g., Holm/Bonferroni) with α = 0.05 (two-sided). Interim analyses are descriptive only. Based on current referral volumes, we anticipate enrolment of ~20–30 patients/year; over five years (N ≈ 100–150), precision for a 10% event rate is ±~6% (95% CI). Analyses will be performed in R; code and analysis manifests will be shared with the de-identified data release. To address time-dependent exposures, treatment status will be modelled as a time-varying covariate using episode splitting, in which each patient’s follow-up is partitioned into consecutive intervals reflecting their actual exposure status at each point in time. Immortal-time bias will be addressed by classifying all person-time prior to treatment initiation as unexposed, ensuring that the pre-treatment observation window is not misattributed to the treated state. Since treatment switches most commonly occurr in the context of clinical progression, they will be handled by opening a new interval at the date of the switch, with exposure updated accordingly to reflect the new treatment, while prior intervals retain their original classification. Treatment discontinuation will be handled analogously, with patients reclassified as unexposed from the discontinuation date onward. Where dose-level data are available, dose changes will be treated by the same mechanism.

We acknowledge two limitations inherent to a routine-care registry setting. First, treatment event dates are recorded at clinical visits rather than to the exact calendar day; interval boundaries therefore carry a degree of temporal imprecision. Second, the frequency and timing of follow-up visits are clinically driven rather than protocol-fixed, which may result in treatment changes between visits not being captured with full precision. To quantify the potential impact of the first limitation, a sensitivity analysis applying a ± 4-week uncertainty window around documented treatment event dates will be conducted. As a further sensitivity analysis, a landmark approach will be applied at 6 and 12 months, classifying patients by treatment status at the landmark and restricting follow-up accordingly; as this approach avoids immortal-time bias by design rather than by statistical adjustment, convergence of results with the primary analysis will serve as an indicator of robustness. Given these constraints, comparative effectiveness estimates derived from time-dependent Cox proportional hazards models should be interpreted as exploratory and hypothesis-generating rather than causal.

### Patient and public involvement

A patient advisory group will contribute to (i) selection and review of PROMs; (ii) assessment of participant burden and visit schedules; and (iii) dissemination of lay summaries. PPI feedback will be documented and reported in future results manuscripts.

## Discussion

This registry study provides a multidisciplinary platform for longitudinal observational data collection in the field of systemic amyloidosis and offers guidance for interdisciplinary diagnostics and care. Patient care, research, and therapy development depend largely on registry studies, especially in rare disease [5,25,26]. Longitudinal registry studies underscore the transformative role of integrating digital medicine resources into research of amyloidosis and other diseases that are considered to be rare. Many other registries, such as the THAOS registry [27,28], the Cardiac Amyloidosis Registry Study (CARS) [29,30], and other local, national, and international registries [31–33] have successfully compiled data volumes that would be unattainable by individual centers alone, thereby enabling study designs that require substantial statistical power. However, this approach may be inherently limited by variations in the scope of data acquisition across participating sites. As noted by the THAOS investigators, variations in the involved and leading specialties at the collaborating centers exist [28], potentially resulting in non-unified sets of examined and supplied variables. These local heterogeneities may limit the unrestricted ability of extrapolation of the collected data.

Barriers to a centralised data collection also include local or national data protection requirements, financial and infrastructural resources, questions of responsibilities, and data access, requiring new strategies for Findable, Accessible, Interoperable, and Reusable (FAIR) data collection and management [34,25]. The ACCB registry study strives towards these principles by providing a digital infrastructure and interoperable data format. By leveraging digital tools, we were able to standardize interdisciplinary data acquisition, thus ensuring high quality data – an essential foundation for research that drives future clinical decision-making. Using such standardised data collection in interoperable formats such as Fast Healthcare Interoperability Resources (FHIR) and Human Phenotype Ontology (HPO) will facilitate integration or joint data analysis together with other local registries, while at the same time following local requirements and constraints. While an important limitation of our registry protocol is the single center design, which is partially owned to the limited scalability of the extensive multidisciplinary care intended, the interoperability of the data format used represents a mitigation strategy to expand research using this protocol beyond the current single-center design and enables pooled, federated analyses. Furthermore, in response to limitations of local registries, innovative models like the central German Portal for Health Data (FDPG) [35] offer promising solutions. By utilizing local digital infrastructure and harmonised data management, FDPG enables interoperability of individual registries – including ACCB – as well as data sharing, while maintaining the decentralised administration. This model ensures the ready availability of data for multicentric research while maintaining high scientific quality by enabling the inclusion of datasets that are specifically suited to address the respective research question. By ensuring rapid availability of comprehensive, interoperable, high-quality data at the ACCB, our approach enhances the efficiency of patient workups while also serving as a reliable resource for longitudinal, including multicentric, analysis – simplifying robust trend and pattern recognition, which is fundamental for new discoveries and innovation.

In comparison to other amyloidosis registries [27, 29, 31,32], the focus of the ACCB registry study lies in the interdisciplinary collaboration of different specialities, as a core foundation of patient characterization and care, including various specialties beyond the “obligatory” cardiological and neurological perspective: Systemic diseases such as amyloidosis, by virtue of their multi-organ involvement, demand a collaborative approach that spans cardiology, neurology, haematology, nuclear medicine, and beyond [1]. By using the Tbase platform [8], the core strength of this registry study lies in its ability to facilitate the seamless acquisition and storage of clinically heterogenous data from different outpatient clinics, minimizing errors resulting from both manual data transfer as well as knowledge gaps in fields outside the scope of an individual’s area of expertise. Our registry has fostered a synergistic environment where specialists from diverse fields contribute to a holistic understanding of amyloidosis pathophysiology and management, improving patient-centred care. This interdisciplinarity is not only crucial for managing systemic diseases but also catalyses innovative research, as novel findings can be rapidly translated into practice across various specialties.

With the ACCB registry, we propose a data collection scheme for different types of amyloidosis that requires relevant resources. Maintaining detailed data collection longitudinally is an important challenge and may lead to loss of data quality in cases of limited resources in the future. Especially regarding the sustainability and scalability of data registries, the definition of minimal data sets is key. With our digital data collection scheme, we hope to enable not only integration of data collection into routine clinical care to save resources in data input and management, but also facilitate patient care for physicians caring for patients with this complex disease by providing a simple interface guiding through important aspects of the disease, as well as a quick overview of relevant patient history, disease course, and patient reported outcomes collected via digital tools.

Looking forward, several future challenges and developments warrant attention. As our registry grows, it will be essential to address the complexities inherent in integrating an ever-expanding array of data types. Incorporating clinical, pathological, laboratory, electrocardiographic, echocardiographic and other imaging variables will require continuous advancements in data management and analytic techniques. Moreover, ensuring data security and maintaining patient privacy in the context of increasingly interconnected digital systems will remain a top priority. Another major challenge is the harmonization of data across different institutions, particularly as CMR or other newer imaging or biomarkers, for example, will expand the diagnostic standard in the future. Standardizing data collection protocols and establishing common definitions will be vital for enabling multicentric collaborations and ensuring the comparability of findings, driving innovative research and the generalizability of the novel findings.

## Conclusion

In conclusion, the ACCB registry serves as a model for the integration of digital medicine into clinical research on complex and rare diseases. By leveraging database software and fostering interdisciplinary collaboration, we have established a robust framework that not only enhances patient care but also lays the foundation for future innovations in the diagnosis and management of amyloidosis. To overcome the challenges ahead, continued investment in digital infrastructure, interdisciplinary collaboration and multicentric partnerships will be crucial to realise the full potential of this approach to improve outcomes for patients with amyloidosis and other complex systemic diseases.

### Strengths and limitations of this study

- This observational registry study protocol provides a structured framework for the clinical management of amyloidosis, encompassing ATTR, AL, and AA subtypes.
- The interdisciplinary design facilitates a comprehensive understanding of the systemic nature of amyloidosis.
- Patient-centred data collection and an interoperable data infrastructure enable robust real-world data analysis and facilitate data sharing.
- Limitations of the protocol include reliance on a multidisciplinary team, and the extensive data requirements—particularly patient-reported outcomes—which depend on patient participation and adherence.
