# Correction: On the crossroads of interdisciplinary medicine in amyloidosis – study protocol for a single-center interdisciplinary registry study

**DOI:** 10.1371/journal.pone.0356916

**Published:** 2026-08-24

**Authors:** Helena Pernice, Gina Barzen, Jakub Piwowarski, Harisa Muratovic-Colic, Anne Pankow, Vera von Landenberg-Roberg, Stephan Bohl, Eva Schrezenmeier, Paul J. Wetzel, Nicolas W. Wieder, Gunnar Fiß, Elisabeth Blüthner, Fabian Knebel, Daniel Messroghli, Stefanie M. Werhahn, Jan Gröschel, Anna-Karina B Maier, Shideh Schönfeld, Christoph Wetz, Jeanette Schulz-Menger, Bettina Heidecker, Axel Nogai, Sebastian Spethmann, Katrin Hahn

There are errors in the author affiliations. The correct affiliations are as follows:

Helena Pernice^1,2^, Gina Barzen^1,4,5,6^, Jakub Piwowarski^1,4,5^, Harisa Muratovic-Colic^1,2^, Anne Pankow^1,7^, Vera von Landenberg-Roberg^1,8^, Stephan Bohl^1,8^, Eva Schrezenmeier^1,9^, Paul J. Wetzel^1,2^, Nicolas W. Wieder^1,2,10^, Gunnar Fiß^1^, Elisabeth Blüthner^1,10,11^, Fabian Knebel^1,12^, Daniel Messroghli^3^, Stefanie M. Werhahn^1,6,13^, Jan Gröschel^1,4,5,6^, Anna-Karina B Maier^1,14^, Shideh Schönfeld^1,14^, Christoph Wetz^1,15^, Jeanette Schulz-Menger^1,4,6,16,17^, Bettina Heidecker^1,6,18^, Axel Nogai^1,19^, Sebastian Spethmann^1,4,5,6^, Katrin Hahn^1,2^

**1** Amyloidosis Center Charité Berlin (ACCB), Charité Universitätsmedizin Berlin, Germany, **2** Klinik für Neurologie mit Experimenteller Neurologie, Charité Universitätsmedizin Berlin, Germany, **3** Medizinische Universität Lausitz - Carl Thiem, Department of Cardiology, Rhythmology and Angiology, Cottbus, Germany, **4** Deutsches Herzzentrum der Charité, Department of Cardiology, Angiology and Intensive Care Medicine, CCM, Berlin, Germany, **5** Charité – Universitätsmedizin Berlin, corporate member of Freie Universität Berlin and Humboldt-Universität zu Berlin, Berlin, Germany, **6** DZHK (German Centre for cardiovascular research), Partner site Berlin, Germany, **7** Department of Rheumatology and Clinical Immunology, Charité-Universitätsmedizin Berlin, Berlin, Germany, **8** Department of Hematology, Oncology and Cancer Immunology, Charité - Universitätsmedizin Berlin, corporate member of Freie Universität Berlin and Humboldt-Universität zu Berlin, Berlin, Germany, **9** Department of Nephrology and Medical Intensive Care, Charité - Universitätsmedizin Berlin, Corporate Member of Freie Universität Berlin and Humboldt- Universität zu Berlin, Berlin, Germany, **10** Berlin Institute of Health at Charité- Universitätsmedizin Berlin, Berlin, Germany, **11** Medizinische Klinik m.S. Hepatologie und Gastroenterologie, Charité Universitätsmedizin Berlin, Campus Charité Mitte, Berlin, Germany, **12** Klinik für Innere Medizin mit Schwerpunkt Kardiologie, Sana Klinikum Lichtenberg, Berlin, Germany, **13** Deutsches Herzzentrum der Charité, Department of Cardiology, Angiology and Intensive Care Medicine, CVK, Berlin, Germany, **14** Department of Ophthalmology, Charité – Universitätsmedizin Berlin, Berlin, Germany, **15** Klinik für Nuklearmedizin, Charité Universitätsmedizin Berlin, Germany, **16** Helios Clinics Berlin Buch, Department Cardiology and Nephrology, Schwanebecker Berlin, Germany, **17** Charité – Universitätsmedizin Berlin, ECRC a joint institution of Charité and MDC, Berlin, Germany, **18** Deutsches Herzzentrum der Charité, Department of Cardiology, Angiology and Intensive Care Medicine, CBF, Berlin, Germany, **19** Onkologische Schwerpunktpraxis Tiergarten, Berlin, Germany.
